# Cross-fusion activates deep modal integration for multimedia recommendation

**DOI:** 10.1371/journal.pone.0327663

**Published:** 2025-07-02

**Authors:** Chong Zhang, ZhiCai Zhang

**Affiliations:** School of Computer and Artificial Intelligence, Beijing Technology and Business University, Beijing, China; Bruno Kessler Foundation: Fondazione Bruno Kessler, ITALY

## Abstract

Recommendation systems play a significant role in information presentation and research. In particular, goods recommendations for consumers should match consumer psychology, speed up product search, and improve the efficiency of product transactions. Online platforms provide product information and interactive information between customers and products. However, the interactive modeling effect of the existing multimedia algorithms on this information must be improved, for instance, by deeply integrating product and interactive information. Accordingly, we propose a cross-fusion-activated multi-modal (CFMM) integration method for recommender systems to achieve deep fusion of product and user information. This method adds a cross-fusion module to fuse the features of different modalities through deep-feature fusion. A fusion loss function is further proposed to improve the recommendation performance of the network. Extensive experiments were conducted on three real-world datasets along with multiple ablation studies to illustrate the effects of the different modules. The experimental results show that the proposed method exhibits better recommendation performance, providing a maximum improvement of 3.8% in the recommendation performance metrics Recall@20, NDCG@20, and Precision@20 in comparisons with existing algorithms. This method realizes a deeper integration of multimodal information; however, the performance can be further improved by extending the multimodal information interaction algorithm to include product and user information.

## Introduction

The large-scale revenues and rapid growth indicate that consumers have a huge demand for e-commerce services. With the development of the Internet and digital technology, information such as images, videos, audio, and text on various modalities in the e-commerce industry has become more abundant, These multimodal datasets contain rich information and can comprehensively describe the characteristics of users and items. Therefore, introducing multimodal data into the recommendation system can help the recommendation system better understand the interests and needs of users and provide more accurate and personalized recommendation services. The method of introducing multimodal information into traditional search engines and recommendation systems is a key issue worthy of extensive research to serve consumers.

Personalized recommendation systems have become indispensable tools for helping users find relevant information from the vast amount of irrelevant content on the Internet. For example, users on e-commerce platforms often select products based on their visual appearance and text descriptions, and both video content and text tags are important for users to find the products they are interested in [1].

Recommendation systems are an effective way to solve the problem of “information overload” [2]. In the 1990s, collaborative filtering technology was first proposed [3], marking significant progress in the field of personalized recommendation systems. Based on the collaborative filtering algorithm, the association rules between users and items have been explored, and many excellent recommendation algorithms have emerged. Rendle et al. [4] proposed matrix factorization (MF) using Bayesian personalized ranking (BPR) loss to directly obtain association information between users and items for recommendation. However, it ignores the large amount of contextual information embedded in multimodal data such as images, texts, and videos. This limitation restricts the ability of MF to capture users’ subtle preferences derived from rich multimodal content. Wang et al. [5] proposed the neural graph collaborative filtering (NGCF) algorithm, which introduces the concept of convolutional layers into the recommendation system, whereby stacking multiple convolutional layers, the high-order connectivity between users and items is identified. However, it still falls short in modeling complex multimodal interactions and fails to fully exploit the semantic and contextual clues present in multimodal data. He et al. [6] proposed light graph convolutional network (LightGCN), arguing that the nonlinear activation function and feature transformation matrix in NGCF have no direct impact on userID/itemID embedding and can be removed. This algorithm reduces the complexity of the model and improves recommendations. But it compromises the model’s ability to capture complex nonlinear relationships within and between modalities, a limitation that hinders its ability to provide highly accurate recommendations based on multimodal data. Wu et al. [7] proposed self-supervised graph learning (SGL), which adds an auxiliary self-supervisory task based on the classic supervised recommendation task, to strengthen node representation learning through self-learning and mine the relevance of long-tailed data. However, it cannot fully utilize the complementary information existing in different modalities, which limits its recommendation performance. Deep learning can automatically learn features of multimodal data, thereby mapping different data to the same latent space and obtaining a unified representation of the data [8]. The general processes of a multimodal recommendation system include multimodal feature extraction, feature interaction, and recommendations [9]. Multimodal features include table features of items and users, descriptive pictures of items, and evaluation text. Feature interaction involves interacting and fusing the representation vectors of different modal features to obtain the representation vectors of items and users. After obtaining the representation vectors of the users and items, the recommendation model is used to calculate the recommendation probability. Among these, the effective fusion of modal representations in different semantic spaces is an important link for improving recommendation accuracy. The feature interactions of multimodal data can be classified into three types: bridging, fusion, and filtering [9]. Bridging refers to the building of a multimodal information transmission channel to enhance the interactive relationship between users and items. Fusion focuses on the multimodal relationships within an item, fusing different multimodal information to generate feature vectors. Filtering involves discarding information that is irrelevant to user preferences and plays a role in removing noisy data. These three methods are frequently used in combination. In addition, different modal representations of the same object contain unique and common semantic information such that distinguishing between them can improve recommendation capability. Therefore, to address the problem of feature differentiation, some studies have proposed disentangled representation learning (DRL) and contrastive learning (CL). DRL is used to deconstruct entangled modal representations as in DICER [10] and MacridVAE [11]. CL is used for modal alignment, to deepen the feature differences between positive and negative samples, as in MICRO [12]. Zhang et al. proposed a multimodal recommendation algorithm, MICRO, which integrates item information graphs. Considering user–item interactions, a bipartite graph was added between items, to capture the semantic similarity between them. A contrastive learning method was also added to compare the feature representations of each modality with the fused feature representation for the fused feature representation to adaptively extract shared information from the multimodal information. The recommendation results showed a significant improvement, but we believe it still has room for improvement in modeling the interactions between multimodal items and users. MICRO mainly focuses on intra-modal and inter-modal feature fusion, ignoring the deeper integration of user preferences and product attributes across modalities. He and McAuley [13] proposed visual Bayesian personalized ranking (VBPR), which uses a pre-trained model to extract the feature representation of multimodal information and adds it to the BPR model to enhance the recommendation capability. But it fails to fully exploit the synergy between different modalities, such as combining visual and textual information in a cohesive way, which limits its ability to provide comprehensive and accurate recommendations. Wei et al. [14] proposed a multi-modal graph convolution network (MMGCN) to represent user-item interaction information and multimodal information as a graph structure, using graph convolution networks for modeling and analysis. Although MMGCN strives to integrate multimodal information, it lacks a strong inter-modal fusion mechanism, resulting in poor performance in capturing the complex relationships between different modalities. Wei et al. [15] proposed a graph refined convolution network (GRCN) that can adaptively adjust the interaction graph decomposition, thereby pruning the noise edges of pseudo-positive interactions. However, it cannot fully solve the problem of multimodal feature integration. GRCN cannot deeply fuse features of different modalities, which limits its effectiveness in providing highly personalized recommendations. Liao et al. [16] proposed a deep multimodal rank learning (DMRL) model to establish a time-related user preference model and combined it with a dynamic sampling strategy based on sorting to improve recommendation accuracy. Liu et al. [17] proposed the PMGT framework, which performs unsupervised pre-training on the graph by considering the multimodal side information of the items and their relationships, thereby learning the representation of the items. Jung et al. [18] proposed the HAT model, which achieves more accurate personalized clothing recommendations by combining consumers’ purchase history information. Yasser Khalafaoui et al. [19] proposed the CADMR framework, which effectively integrates and utilizes heterogeneous multimodal data by combining cross-attention mechanism and disentanglement learning. Wu et al. [20] proposed a multimodal news recommendation algorithm (MM-Rec), which extracts ROIs from news images through mask-region-based convolutional neural network (RCNN) and then used the pre-trained model vision-and-language BERT (ViLBERT) to encode text and image ROIs and model their intrinsic correlation. Liu et al. [21] recently proposed a gated recurrent unit (AGRU) model based on attention mechanism. The model effectively captures the sequence characteristics and long-term dependencies of well logging data through the attention mechanism, and realizes flexible and context-dependent weighting of different parts of the sequence. Tao et al. [22] proposed the use of a graph attention network (MGAT) on a multimodal information interaction graph to capture complex interaction patterns hidden in user behavior. Graph Attention Network itself has high computational complexity, especially when dealing with large-scale graph structures. Therefore, MGAT may face challenges in computational efficiency when dealing with large-scale datasets. Sun et al. [23] proposed a multimodal knowledge-graph self-attention network (MKGAT). MGAT and MKGAT are highly dependent on large amounts of high-quality multimodal data. If the data is insufficient or noisy, the performance of the model may be affected. Qi et al. [24] proposed a POI category recommendation model (PPCM) based on group preference, which protects user privacy and classifies similar users through LSH technology, combines LSTM and attention mechanism to capture user dependencies, and uses POI categories to mine user interests, thereby improving recommendation performance. Although the MICRO method takes into account the importance of various modal features and promotes the integration of modalities through contrastive learning and other methods, but we believe it still has room for further improvement in establishing the interaction between multimodal items and users and in multimodal feature interaction.

Based on previous studies, we believe that the main limitations of current methods are the insufficient feature interactions between modalities and the inability to deeply integrate the fused multimodal information and the interactions between users. To address these challenges, we propose a cross-fusion activation deep modal integration method for multimedia recommender systems (CFMM). Our method aims to achieve a deeper fusion of product and user information by introducing a Cross-Attention module, which integrates item features from different modalities and user-item interactions through deep feature fusion. In addition, we design a fusion loss function to optimize the multimodal feature embedding process, ensuring that the fused representation better reflects the user preferences and item features.

The main innovation of our work lies in the development of the Cross-Attention module and the fusion loss function, which together enhance the ability of recommender systems to deeply integrate multimodal information. By addressing the limitations of existing methods, our CFMM approach helps advance the state of the art in multimodal recommender system algorithms.

**Fig 1:**
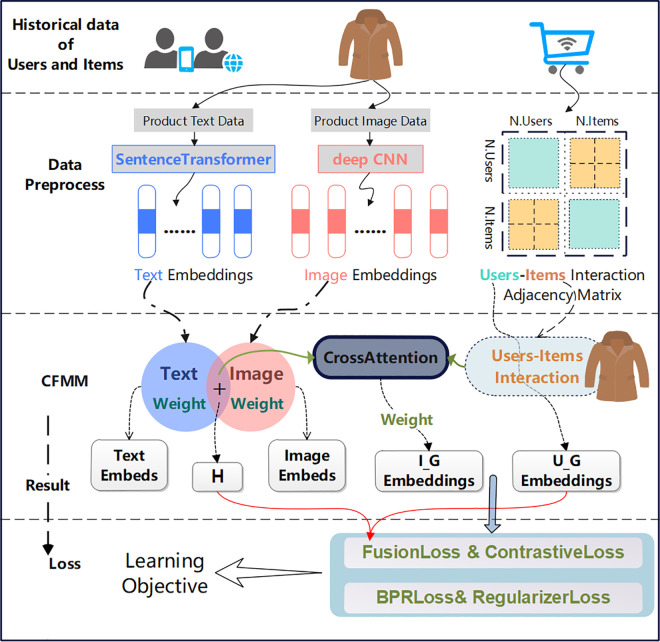
CFMM model framework.

As shown in Fig 1, the CFMM algorithm is an algorithm that focuses on improving the performance of multimedia recommendation systems. It does this by deeply processing the basic data of users and products, product text descriptions, and product image information. First, the algorithm uses SentenceTransformer and deep CNN to extract the text and image features of the product respectively and generate the corresponding embedding vectors. Then, the product review information is used to construct the interaction adjacency matrix between users and products to capture the complex relationship between users and products. The Cross-Attention module dynamically focuses on the most important parts of the two inputs by calculating the attention weights, thereby achieving deep feature fusion. The module receives two types of inputs: one is the item embedding representation (I_G_Embeddings) containing user features and user-item interaction relationships; the other is the item embedding representation (H) that fuses text and image features. During the training process, the algorithm uses comprehensive losses, including BPR loss, Fusion loss, Regularization loss, and Contrastive loss, to optimize model parameters by minimizing these losses and improve recommendation accuracy. Finally, the CFMM algorithm outputs optimized user and product embedding representations, and generates personalized recommendation lists based on these representations.

Multimodal information improves recommendation accuracy, but the fusion method needs to be improved. This study introduces a cross-fusion activated multimodal ensemble (CFMM) for recommendation. Main contributions: 1) The cross-attention fusion model deepens the interaction between multimodal items and users. 2) The fusion loss optimizes the multimodal feature embedding of items. 3) The CFMM integrates four modal loss functions.

The remainder of this paper is organized as follows: The feature enhanced multimodal recommendation method is introduced in Section 2. The experimental details and dataset preparation are presented in Section 3. The recommendation results of the different methods and ablation studies on Amazon public datasets are presented and analyzed in Section 4. The conclusions of this study are summarized in Section 5.

## Methodology

### Overall structure of CFMM method

A recommendation method that combines multimodal information for e-commerce product recommendations was proposed by Zhang et al. [12]. They calculated the similarity of text and image information between products, thereby constructing a product similarity graph and combining it with the user-item interaction graph using different collaborative filtering models, to complete product

**Table:** 

Algorithm CFMM ** Require:** ** Adj :** User-Item Interaction Adjacency Matrix ** Build Item Graph :** Boolean. Default is False **Ensure:** ** U_G_Embeddings :** Contains User Features and Users-Items Interaction ** I_G_Embeddings :** Weighted ( Item Features, Users-Items Interaction, Text and Image Fusion of Items, Items-Items, Correlation) ** Image_Embeds :** Item Features that Fuse Image Similarities ** Text_Embeds :** Item Features that Fuse Item Similarities ** H :** Items Embedding that Fused Image and Text Features **Forward** 1: Image_Feats ← Linear( Pretrained Image Features ) 2: Text_Feats ← Linear( Pretrained Text Features ) /* Image/Text_Feats after dimensionality reduction of original data */ 3: **if** Build_Item_Graph **then** ▷The first batch the value is True 4: Image_Adj ← Building a kNN weighted graph( Image_Feats ) 5: Image_Adj ← ( 1 – λ_*coeff* ) · Image_Adj + λ_*coeff* · Original_Adj 6: Text_Adj ← Building a kNN weighted graph( Text_Feats ) 7: Text_Adj ← ( 1 – λ_*coeff* ) · Text_Adj + λ_*coeff* · Original_Adj 8: **else** 9: Image_Adj ← Image_Adj.detach( ) 10: Text_Adj ← Text_Adj.detach( ) 11: **end if** /* Image/Text_Adj is a kNN adjacency graph, which is the top k most relevant Images/Texts between Items */ 12: Image_Embeds ← sparse.mm( Image_Adj, ItemId ) 13: Text_Embeds ← sparse.mm( Text_Adj, ItemId ) /* ItemId features that fuse Image/Text similarities */ 14: Weight ← Softmax( Cat([ Query( Image_Embeds ), Query( Text_Embeds ) ]) ) /* The attention Weight reflects the importance of image and text information for each item */ 15: H ← Weight[: , 0] · Image_Embeds + Weight[: , 1] · Text_Embeds /* Weighting Image/Text relevance between items */ 16: Adj_Embeddings ← mean( sparse.mm( Adj, UserAndItemId ) ) /* Adj_Embeddings is composed of multiple layers of graph convolution to capture more complex and deeper interactive relationships */ 17: U_G_Embeddings, I_G_Embeddings ← Split( Adj_Embeddings, [ Nusers, Nitems ] ) /* Its not only contain user and item features, but also the interactions between users and items */ 18: H_norm ← F.normalize( H ) 19: Weight ← Softmax( CrossAttention( I_G_Embeddings, H_norm ) ) 20: I_G_Embeddings ← Weight[: , 0] · I_G_Embeddings + Weight[: , 1] · H_norm /* It is a representation that combines multiple information sources and performs personalized weighting according to attention weights */ 21: **return** U_G_Embeddings, I_G_Embeddings, Image_Embeds, Text_Embeds, H

recommendations. This study adopts this idea and believes that there is still room for improvement in the fusion of different modal and user-item information features. Accordingly, a cross-fusion multimodal feature fusion algorithm is proposed for a multimodal product recommendation system.

**Fig 2:**
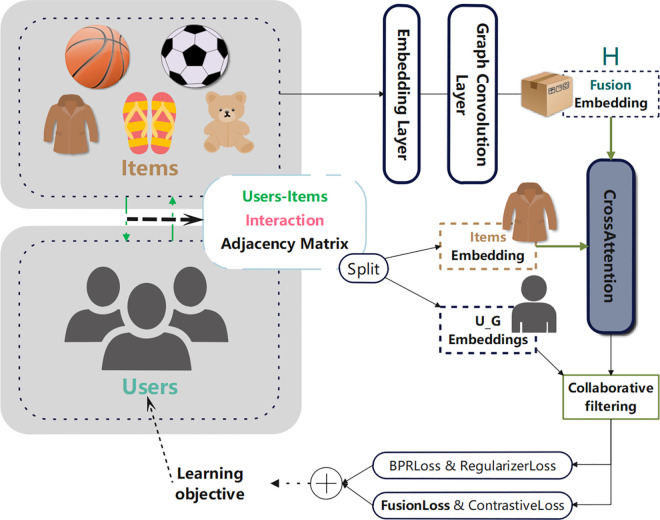
CFMM method. The CFMM method diagram comprehensively presents a complete system that builds an adjacency matrix through product text and image features combined with user-item interaction information, uses the Cross-Attention module to achieve deep fusion of multimodal features, and optimizes the model through BPR loss, Fusion loss, Regularization loss and Contrast loss functions, and finally outputs optimized user and item embedding representations to improve the performance of the multimedia recommendation system.

The framework diagram of the CFMM algorithm is shown in Fig 2, which mainly shows the core components and processes of the algorithm. The Embedding Layer is responsible for mapping the original IDs of users and items as well as image and text features into low-dimensional dense vectors. Graph Convolution integrates the item embeddings of text and image into the similarity graphs of text and image respectively, and updates the embedding representation using the interaction relationship between users and items. The Fusion part fuses the image and text embeddings by calculating the attention weights to obtain the fused item embedding representation H. The Split part is used to separate the adjacency matrix with the interaction relationship between users and items into the item embedding representation with the interaction relationship and the user embedding representation with the interaction relationship. The Cross-Attention part focuses on calculating the importance between the fused multimodal item features H and the item features (Items Embedding) with the interaction relationship between users and items, and then updates the item embedding representation according to the calculated weights, thereby emphasizing whether to pay more attention to the interaction relationship between users and items or the similarity between items. Finally, during the network training phase, a loss function fusion based on fused feature loss is designed and added to better capture the complex relationship between users and items and the correlation between multimodal features, so as to recommend the learned products to users.

### Construction of item-item adjacency matrix

The cross-fusion-based multimodal recommendation method follows the concept of Zhang et al. [12]. The text and image information of the items, as well as the user-item interaction information, are embedded in the representation vector. To explore the correlation between items, the cosine similarity between the multimodal features of the items is calculated. A sparse graph adjacency matrix is constructed using k-nearest neighbor (kNN) [25], and the final similarity adjacency matrix is normalized [12]. The cosine similarity matrix $S^{m}$ is calculated as follows:


$$S_{ij}^{m}=\frac{{\left(e_{i}^{m}\right)}^{T}e_{j}^{m}}{\left\|e_{i}^{m}\right\|\left\|e_{j}^{m}\right\|}$$ (1)

to obtain the similarity matrix ${\tilde{S}}^{m}$ after subjecting the matrix to kNN sparse processing and normalization. The raw modality features $e_{i}^{m}$ are transformed into high-dimensional features ${\tilde{e}}_{i}^{m}$ and the transformation process is added to the network training. This algorithm learns autonomously to extract effective features. These steps were repeated to obtain the similarity adjacency matrix ${\tilde{A}}^{m}$ for the trained items. However, Zhang et al. [12] believed that the similarity adjacency matrix ${\tilde{S}}^{m}$ constructed from the original features still carries rich information on the initial graph structures. Therefore, the skip connection method was used to superimpose the original similarity adjacency matrix onto the trained adjacency matrix to stabilize the training process. The adjacency matrix of the multimodal information for an item can be obtained using (2).


$$A^{m}=\lambda {\tilde{S}}^{m}+(1-\lambda ){\tilde{A}}^{m}$$ (2)

where λ∈(0,1) is the proportional coefficient of the skip connection. It is a hyperparameter used to adjust the proportions of the original features, and $A^{m}$ is the final adjacency matrix of modality m. After obtaining the item adjacency matrix $A^{m}$, we used the methods of Zhang et al. [12], Wu et al. [26] and He et al. [6] and stacked multiple graph convolutional layers to capture the high-order item-item relationships. In the lth layer, the adjacency matrix is calculated using (3).


$$H_{\left(l\right)}^{m}=A^{m}H_{\left(l-1\right)}^{m}$$ (3)

where $H_{\left(l\right)}^{m}$ is the lth layer item-embedding matrix of modality m. The ith row of the matrix represents the embedding vector for item i. The initial input embedding matrix $H_{\left(0\right)}^{m}$ is the ID embedding vector of the items for all modalities m∈M. Thus, $H_{\left(l\right)}^{m}$ represents the high-order item-item embedding matrix of modality m obtained after l layers of mapping, as shown in the H output by fusion embedding, in Fig 2.

### Cross-attention module

The Cross-Attention module is an attention mechanism that allows the model to dynamically focus on the most relevant part of one sequence when processing two different sequences. It mainly plays the role of deeply integrating multimodal features in the recommendation system. In addition to multimodal feature fusion, Cross-Attention can also consider the interaction information between users and items. It calculates attention weights and dynamically focuses on the correlation between items features and user-item interaction features. By modeling the interaction between user features and multimodal features of items, the model can capture users’ preferences for different aspects of items. The Cross-Attention mechanism helps the model capture these complex relationships by deeply fusing multimodal features and user-item interaction information. This fusion not only considers the attributes of the item itself, but also incorporates the interaction information between users and items, allowing the recommendation system to more comprehensively understand user interests and item characteristics, and thus provide more accurate and personalized recommendation services. In this study, the Cross-Attention module is used to fuse two different types of information: an embedding representation containing the original item ID features and the user-item interaction relationship, and an embedding representation containing the item’s text, image information, and the correlation between items.

#### Input preparation.

**Fig 3:**
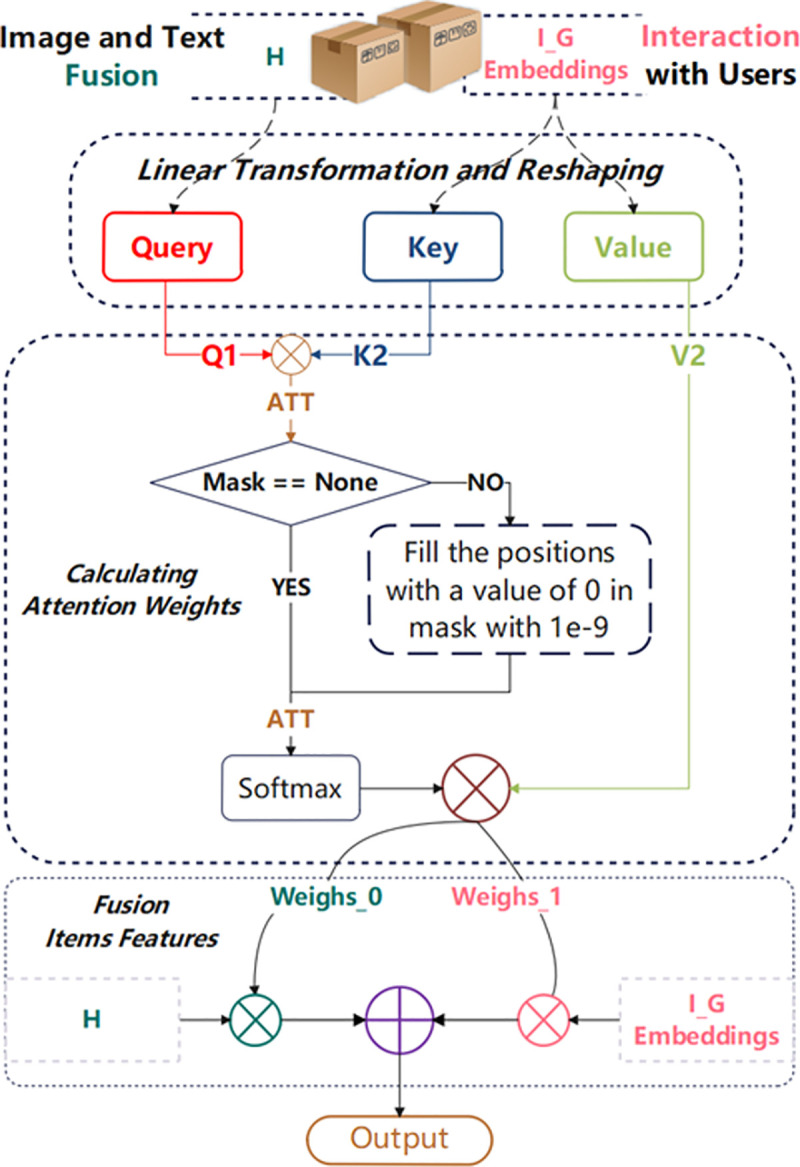
Cross-attention module network structure. Cross-attention module network structure and working principle used for product feature fusion, including input preparation, projection layer, scaled dot product attention, softmax normalization, and weighted sum of values.

As shown in Fig 3, the Cross-Attention module receives two tensors as inputs: I_G_Embeddings and H. The I_G_Embeddings is the item embedding representation enhanced by the multi-layer graph convolution process. It not only contains the characteristics of the item itself, but also integrates the information of users and items that have interactive relationships with the item, so as to more comprehensively represent the characteristics of the item in the recommendation system. By contrast, H represents the comprehensive representation obtained by fusing image and text features. First, the original image and text embedding vectors are reduced from higher dimensions (4096 and 1024) to 64 dimensions through dimensionality reduction operations. For the reduced image and text features, k-nearest neighbor (k-NN) [25] graphs are constructed respectively, which represent the similarity relationship between image and text features. Then, the k-NN graphs of image and text are weighted fused respectively, and the original k-NN graphs are fused. The weight of weighted fusion is controlled by lambda_coeff. The purpose of this step is to combine the characteristics of the original graph and the processed graph to obtain more comprehensive similarity information. Next, the k-NN graphs of image and text are used to update the item embedding representation. Through multi-layer graph convolution operations, the similarity information of image and text is integrated into the item embedding representation. After that, the item embedding representations of image and text are fused, and the attention scores of each item on the image and text are calculated. The scores are converted into probability distributions using the softmax function. These probability distributions reflect the importance of image and text information to each item. Finally, according to the attention weights, the item embedding representations of image and text are weighted and added to obtain the final fused representation H. This representation takes into account both image and text information and weights them according to their importance. However, before applying it to the cross-attention module, this H must be further normalized by the L2 operator to ensure that all feature vectors are located on the unit modulus. This helps in the stable calculation of the subsequent attention fusion unit.

#### Cross-attention for product information fusion.

The core formula of cross-attention is based on a standard attention mechanism [27]. However, we focus on cross-modal feature fusion. As shown in Fig 3, given the user-item interaction embedding containing user features and the item embedding fused with image and text features, the cross-attention mechanism is calculated as follows:


$$\begin{array}{c} CrossAttention(I,H)=softmax(\frac{I\cdot W_{Q}\left(H\cdot W_{K}\right)}{\sqrt{d_{k}}})H\cdot W_{V}\cdot W_{O}\\ Fused\_items=CrossAttention[0]\cdot I+CrossAttention[1]\cdot H \end{array}$$ (4)

where $I\in R^{b\times s_{1}\times d_{1}}$ is a tensor containing user features and user-item interactions, $H\in R^{b\times s_{2}\times d_{2}}$ is a fusion tensor of item text features and image features. b is the batch size, $s_{1}$ is the sequence length, $d_{1}$ is the feature dimension, $d_{k}$ is the dimension of the key vector used to scale the dot product to prevent the gradient from vanishing. $W_{Q}$ is the query projection matrix, $W_{K}$ is the key projection matrix, $W_{V}$ is the value projection matrix, and $W_{O}$ is the output projection matrix. In (4) projects the input tensor I and tensor H into the query space and key space respectively, then the dot product of the query and the key is calculated and scaled by $d_{k}$, apply the softmax function to calculate the attention weights, the values are weighted summed using the attention weights. The final result is the attention weight calculated by the Cross-Attention mechanism. Then, the two sets of features are fused according to the calculated weights, which can get the final fusion item characteristics Fused_items.

**Projection Layer:** The input tensors I and H, are projected onto the query, key, and value spaces using separate linear layers. The H is treated as a key (K) and value (V), whereas I are projected as a query (Q). The projected tensors are then reshaped.

**Scaled Dot Product Attention:** The attention weights were calculated by taking the scaled dot product between query (Q) and key (K).

**Linear Layer Output Cross Weights:** The cross-products were further projected through another linear layer to obtain a cross-weighted representation for use in the recommender prediction or ranking task.

**Weighted Sum of Values:** Then cross-attention weights were then used to calculate the I and H, The weighted sum of the two sets of product feature vectors was used to obtain the final output of the cross-attention module. The weight of this output is determined by their cross-attention calculation results.

### Loss function

In this study, to optimize the performance of the recommendation model, we designed a comprehensive loss function that integrates multiple loss terms, including BPR [4], fusion, regularization, and contrastive loss [12].

**BPR Loss:** The Bayesian personalized ranking loss [4] is a commonly used loss function in recommendation systems for measuring the prediction accuracy of the interaction strength between user and item embeddings. This is mathematically expressed in (5):


$$BPR\_Loss=\lambda_{B}\times (-\frac{1}{\left|\beta \right|}\sum_{(u,i^{+},i^{-})\in \beta }\log\sigma (user_{u}^{T}pos\_item_{i^{+}}-user_{u}^{T}neg\_item_{i^{-}}))$$ (5)

where β represents the training batch; u represents the user index; $i^{+}$ and $i^{-}$ represent the indexes of positive and negative items, respectively; σ represents the sigmoid function; $user_{u}$,$pos\_item_{i^{+}}$ and$neg\_item_{i^{-}}$ represent the embedding vectors of user u, positive item $i^{+}$, and negative $i^{-}$, respectively; $user_{u}^{T}pos\_item_{i^{+}}$ represents the preference score of user u for a positive sample item $i^{+}$; $user_{u}^{T}neg\_item_{i^{-}}$ represents the preference score of user u for a negative sample item $i^{-}$.

**Fusion Loss:** In recommendation systems, the quality of embedding vectors (such as user and item embeddings) is crucial to the performance of the model [28]. Although traditional matrix decomposition methods are effective, they often fail to capture the complex relationships between embedding vectors and the effects of multimodal information fusion [29–31]. As for the Fusion Loss, we are inspired by the BPR Loss, which constructs the loss function by calculating the relative positions of items in the user preference ranking, avoiding the predicament of lacking accurate item preference labels. This design cleverly bypasses the reliance on specific labels and instead focuses on the relative ranking relationship between items, thereby effectively optimizing the ranking performance of the recommendation system. Based on this idea, we innovatively proposed Fusion Loss. Fusion Loss also uses the core concept of relative ranking, but further focuses on the role of fusion features in product ranking. Specifically, we calculated the text and image features of the items, using ranking feedback from user preferences to guide the network in extracting more expressive features for the items. Specifically, Fusion Loss finds out the products that consumers like and dislike from the fusion features representing the products, so that the fusion features of the products that consumers like are ranked first, and the fusion features of the products that consumers dislike are ranked last, and finally finds out the products preferred by consumers from the fusion features of the products.

In this study, Fusion Loss was specifically designed for recommendation systems. It focuses on measuring the contribution of multimodal item fusion information for recommendation accuracy. In the recommendation system, Fusion Loss encourages users and their interacted items to be clustered more closely in the embedding space, while keeping a certain distance from items that have not interacted in the embedding space. We call the items that the user has interacted with positive samples, and the items that the user has not interacted with negative samples. By increasing the distance between positive and negative samples, the fusion accuracy of multimodal information is improved, thereby improving the quality of recommendations.

**Fig 4:**
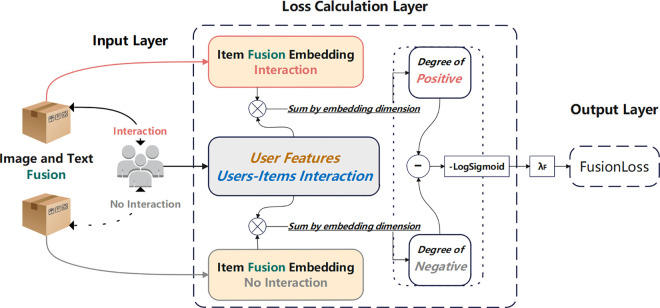
Schematic of Fusion Loss calculation framewor. To optimize interaction prediction accuracy between users and multimodal fusion item information.

As shown in Fig 4, Fusion Loss first receives the fused product features and the user embedding vector of the user-item interaction relationship through the input layer. Then in the interaction calculation layer, the dot product of the user embedding vector and the positive/negative item fusion embedding vector is calculated to obtain the positive/negative interaction score, which reflects the user’s potential interest in different items. In the loss calculation layer, the difference between the positive interaction score and the negative interaction score is transformed using the LogSigmoid function to obtain the loss value, and the final Fusion Loss is obtained by taking the negative average. By minimizing Fusion Loss, on the one hand, the influence of the fusion product on the user’s purchase intention can be integrated, and on the other hand, the model can be constrained to accurately extract the image and text features of the product, thereby improving the performance of the recommendation system.

The core idea is to optimize the model by penalizing the difference in dot product scores between user and fused item embeddings. Specifically, we calculate the difference in dot product scores between user embedding and fused positive and negative item embeddings, and balance their contribution to the total loss by multiplying them by a small coefficient, which is mathematically expressed in (6):


$$Fusion\_Loss=\lambda_{F}\times (-\frac{1}{\left|\beta \right|}\sum_{(u,i^{+},i^{-})\in \beta }\log\sigma (user_{u}^{T}h_{i^{+}}-user_{u}^{T}h_{i^{-}}))$$ (6)

where $h_{i}$ represents the fusion embedding vector of item i; $\lambda_{F}=10^{-5}$ is the weight coefficient of Fusion Loss, which is used to balance the impact of different loss functions on network training.

In minimizing this loss function, the expectation is that the user u ‘s preference score for the positive sample item $i^{+}$ will be as high as possible compared with the preference score for the negative sample item $i^{-}$, thereby optimizing the embedding vectors of users and items, especially the fused item embedding vector $h_{i}$, for the recommendation systems to predict user preferences more accurately.

**Regularization Loss:** As a commonly used technique in recommender systems, Regularization Loss prevents model overfitting and improves generalization ability [32]. Overfitting usually occurs when the model complexity is so high that the model starts to learn the noise in the training data, rather than the true data pattern. Regularization avoids overfitting by imposing a penalty term on the model parameters to confine the complexity of the model [33]. which is mathematically expressed in (7):


$$Regularization\_Loss=\lambda_{R}\times (\frac{1}{2\left|\beta \right|}({\left\|users\right\|}_{2}^{2}+{\left\|pos\_items\right\|}_{2}^{2}+{\left\|neg\_items\right\|}_{2}^{2}))$$ (7)

where ${\left\|.\right\|}_{2}$ represents the L2 norm; decay is a decay factor used to control the weight of the regularization term; ${\left\|users\right\|}_{2}^{2}$ is the L2 norm sum of squares of user-embedding vectors; ${\left\|pos\_items\right\|}_{2}^{2}$ is the L2 norm sum of squares of positive-item embedding vectors; ${\left\|neg\_items\right\|}_{2}^{2}$ is the L2 norm sum of squares of negative-item embedding vectors, and the average value is multiplied by $\lambda_{R}$ to control the weight of the regularization term.

**Contrastive Loss:** A loss function proposed by Zhang et al. [12], contrastive loss constrains multimodal information fusion. This study applies contrastive loss to the recommendation system to improve the model’s ability to capture item features (such as images and text) and enhance the prediction accuracy of user-item interactions. This is mathematically expressed in (8):


$$Contrastive\_Loss=\lambda_{C}\times (-\frac{1}{\left|\beta \right|}\sum_{i\in \beta }\log(\frac{\exp(sim(z_{i},z^{+})/\tau )}{\sum_{i\in \beta }\exp(sim(z_{i},z_{j})/\tau )}))$$ (8)

where $z_{i}$ is the current sample in the batch; $z^{+}$ is the positive sample corresponding to $z_{i}$; $sim(z_{i},z^{+})$ is the similarity between the current sample $z_{i}$ and the corresponding positive sample $z^{+}$;τ is a parameter used to control the smoothness of the similarity score; and $\lambda_{C}$ is used to control the effect of contrast auxiliary tasks.

The loss function used in this study is expressed as follows:


$$TotalLoss=\lambda_{B}*BPRLoss+\lambda_{R}*RegularizationLoss+\lambda_{C}*ContrastiveLoss+\lambda_{F}*FusionLoss$$ (9)

## Experiments

### Data preprocessing

This experiment uses Amazon Product datasets, including (a) Clothing, Shoes and Jewelry, (b) Sports and Outdoors, and (c) Baby [12,34]. These datasets [35] include 4096-dimensional product image data, 1024-dimensional product information fields, and user-product interaction information. The product information field uses the content of the title, descriptions, categories, and brand fields.

First, we read the compressed JSON file and store the metadata and user-item interaction data in an uncompressed JSON file. We extract unique user and item IDs from the review data, assign different identifiers to them, and establish a mapping relationship between users, items, and their respective IDs. This mapping can be used to construct a user-item interaction dictionary to obtain a normalized adjacency matrix suitable for graph neural networks. The adjacency matrix clarifies the interaction relationship between users and items, providing key information for subsequent user behavior analysis and recommendation system modeling.

In addition, we randomly matched the products that each user had reviewed (positive-pair) with the user-item that had no interaction (negative-pair), and divided them into 80% training set, 10% validation set, and 10% test set. The SentenceTransformer [36] library and the pre-trained BERT model were used to efficiently extract feature vectors from text data. We also performed data cleaning and read the pre-extracted product image features from binary files. If a product did not have image features, the average of all product image features was used as a substitute. This ensured that each product had a corresponding image feature vector, thereby maintaining data integrity and consistency across the dataset.

Amazon product data was ultimately converted into user-item interaction data, product text features, and product image features.

### Baselines

To evaluate the effectiveness of the proposed model, we compared it with multiple state-of-the-art recommendation models. These baseline models are divided into collaborative filtering methods (i.e., ItemKNN, MF, NGCF, LightGCN, and SGL) and deep content-aware recommendation models (i.e., VBPR, MMGCN, GRCN, and MICRO).

- ItemKNN [37]: An item-based collaborative filtering method that recommends items to users by calculating the similarity between items.
- MF [4]: A matrix factorization method that uses Bayesian personalized ranking (BPR) loss to optimize the user-item interaction matrix, utilizing only direct user-item interaction information.
- NGCF [5]: Neural graph collaborative filtering learns embedded representations of users and items by constructing a user-item bipartite graph and utilizing a graph convolutional network to capture the high-order connectivity between users and items.
- LightGCN [6]: A lightweight GCN simplifies the design of the GCN, removes feature conversion and nonlinear activation, and retains only two core components: graph convolution and layer combination, which effectively improves recommendation performance.
- SGL [7]: Self-supervised graph learning enhances node representation learning by generating multiple views of a node and maximizing the consistency between different views of the same node.
- VBPR [13]: Based on the BPR model, the visual features and ID embeddings of the items are combined and input into the matrix factorization framework as a representation of the items to improve recommendation performance.
- MMGCN [14]: A multimodal graph convolutional network that builds a specific user-item interaction graph for each modality, captures modality-specific user preferences and item representations through graph convolution operations, and finally aggregates all modality-specific representations to predict user and item interactions.
- GRCN [15]: Graph-refining convolutional network that refines the user-item interaction graph in multimodal recommendation systems by identifying pseudo-positive feedback and pruning the corresponding noisy edges.
- MICRO [12]: Latent structure mining and contrastive modality fusion method mines the latent item relations hidden in multimodal features and designs a multimodal contrast framework to promote fine-grained multimodal fusion, thus enhancing the recommendation effect.

### Evaluation protocols

To rigorously evaluate the performance of our multimedia recommendation model, we adopt three main evaluation metrics: Recall@K [38,39], NDCG@K [40–42], and Precision@K [2,43] where K is set to 20. These metrics provide comprehensive insights into the recall, ranking accuracy, and precision of the model, respectively.

The sampling process is carefully designed to ensure the balance and representativeness of the dataset. Users are randomly sampled from the existing user base, and for each sampled user, a positive item (Interacted items) and a negative item (uninteracted item) are randomly selected from the user’s interaction history. Ensuring that each user has exactly one positive interaction item and one negative interaction item is critical to maintaining a balance between positive and negative samples during evaluation.

By leveraging these evaluation metrics and the carefully designed sampling process, we are able to conduct a comprehensive and rigorous evaluation of the performance of our multimedia recommendation model.

### Implementation details

The PyTorch framework [44] was used to build our model, setting the embedding dimension of all the models involved in the comparison to 64. The models were trained using the AdamW optimizer [45], and the batch size was fixed at 1024. The Xavier initialization strategy [46] was used to initialize the model parameters and promote effective learning of the model. To determine the optimal hyperparameter combination, a detailed grid search was performed on the validation set. The learning rate was fine-tuned to 0.0005. In the kNN sparsification step, the k value was set to 10 to balance information retention and computational efficiency. In addition, the Lambda Coefficient λ of the skip connection was set to 0.8 to find the best balance between preserving the original graph structure information and introducing the learned graph structure information. The temperature parameter τ was set to 0.5 to adjust the similarity calculation in the contrastive loss. The weight coefficient $\lambda_{B}$ of the BPR loss was set to 1; the weight coefficient $\lambda_{R}$ of the Regularization Loss was set to 1*10^−5^; the weight coefficient $\lambda_{C}$ of the Contrastive Loss was set to 0.03; and the weight coefficient $\lambda_{F}$ of the Fusion Loss was set to 1*10^−5^. To avoid overfitting of the model during training, an early stopping strategy was adopted such that if the Recall@20 metric on the validation set failed to improve within 10 consecutive training cycles (epochs), the training process was terminated early. This strategy ensures the generalization ability of the model while reducing the unnecessary consumption of computing resources.

## Results

### Performance comparison

In this section, we comprehensively compare the performance of the proposed CFMM model with those of the baseline MICRO and other advanced methods. Specifically, we focus on evaluating the overall performance under different categories to demonstrate the effectiveness and improvement provided by CFMM.

Table 1 presents the overall performance comparison results of the proposed CFMM model with various state-of-the-art multimedia recommendation methods and other collaborative filtering (CF) baselines on three real datasets.

**Table 1:** Comparison of performance metrics between the baseline model and CFMM on three datasets.

Model	Clothing			Sports			Baby		
	R@20	P@20	NDCG@20	R@20	P@20	NDCG@20	R@20	P@20	NDCG@20
ItemKNN	0.0280	0.0014	0.0131	0.0410	0.0022	0.0212	0.0317	0.0017	0.0152
MF	0.0191	0.0010	0.0088	0.0430	0.0023	0.0202	0.0440	0.0024	0.0200
NGCF	0.0387	0.0020	0.0168	0.0728	0.0038	0.0332	0.0591	0.0032	0.0261
LightGCN	0.0470	0.0024	0.0215	0.0803	0.0042	0.0377	0.0698	0.0037	0.0319
SGL	0.0598	0.0030	0.0268	0.0905	0.0047	0.0412	0.0745	0.0040	0.0328
VBPR	0.0481	0.0024	0.0205	0.0582	0.0031	0.0265	0.0486	0.0026	0.0213
MMGCN	0.0501	0.0024	0.0221	0.0638	0.0034	0.0279	0.0640	0.0032	0.0284
GRCN	0.0631	0.0032	0.0276	0.0833	0.0044	0.0377	0.0754	0.0040	0.0336
MICRO	0.0782	0.0040	0.0351	0.0988	0.0052	0.0457	0.0892	0.0047	0.0402
**CFMM**	**0.0796**	**0.0040**	**0.0355**	**0.1012**	**0.0054**	**0.0465**	**0.0897**	**0.0047**	**0.0407**
**△Improvement**	**1.8%**	**0.0%**	**1.1%**	**2.4%**	**3.8%**	**1.8%**	**0.6%**	**0.0%**	**1.2%**

△Improvement represents the relative improvement percentage of the CFMM over the best baseline.

The evaluation indicators included Recall@20, NDCG@20, and Precision@20, where K was set to 20.

• The CFMM model significantly outperformed all baseline methods on all datasets, demonstrating the effectiveness of the proposed method. Specifically, CFMM improved the Recall@20 indicator on the Clothing, Sports, and Baby datasets by 1.8%, 2.4%, and 0.6%, respectively, compared with those of the strongest baseline method. These results demonstrate that the CFMM is suitable for multimedia recommendation tasks because it deeply integrates cross-modal information and item relationships.

• By comparing the performance of the CFMM algorithm and the MICRO algorithm on three real data sets, we found that the improvement of CFMM in the three indicators of Recall@20, NDCG@20 and Precision@20 is statistically significant compared with the MICRO algorithm. The P-value is 0.0312. This P-value is much lower than the commonly used significance level (such as 0.05), indicating that the performance improvement of the CFMM algorithm is not accidental, but has statistically reliable evidence.

### Performance comparison of different learning rates

In this section, we used three real-world datasets, Clothing, Sports, and Baby, to train the CFMM model, and set three different learning rates (0.005, 0.0005, and 0.00005) to observe the specific impact of the learning rate on the model performance.

Three different learning rates (0.005, 0.0005, 0.00005) were selected to explore the impact of learning rate on model convergence speed, training stability and generalization ability. A larger learning rate (0.005) can converge quickly but is prone to overfitting, a smaller learning rate (0.00005) converges slowly and has poor learning effects, and the middle value (0.0005) achieves the best balance between convergence speed and model performance, ensuring stable training and good generalization ability.

**Fig 5:**
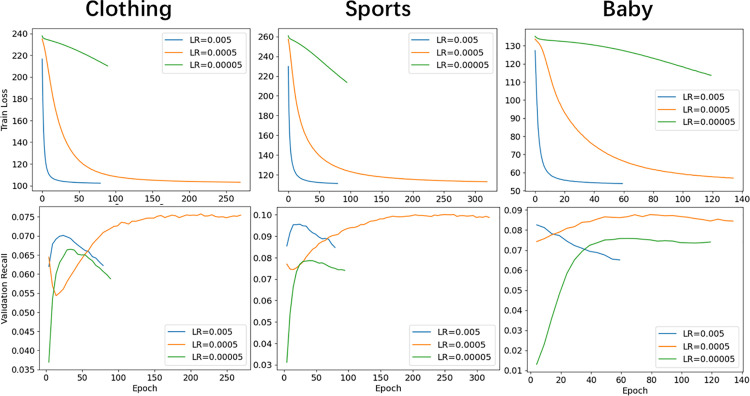
Performance comparison of different learning rates. The performance of the CFMM method in terms of training loss and validation recall under three different learning rates.

As shown in Fig 5, in order to comprehensively evaluate the performance of the model under different learning rates, in this section we use training loss and Recall@20 as the main performance evaluation indicators. Training loss is used to measure the degree of fit of the model, and Recall@20 is used to measure the proportion of products that users have actually interacted with among the top 20 recommended products.

#### Training loss analysis.

The following is a detailed analysis and conclusion summary of the training loss performance of CFMM in Fig 5 under three different learning rates.

**Learning Rate is 0.005:** The training loss of the CFMM method on the three datasets all began to decline rapidly within 20 epochs. Due to the fast convergence speed, the algorithm completed the training within 100 epochs, and the final training loss value was relatively small. However, despite the good performance at this learning rate, there may be a risk of overfitting, because too fast convergence may cause the model to perform well on the training data, but poor generalization ability on the test data.

**Learning Rate is 0.0005:** The training loss of the CFMM method on the three datasets all steadily decreased within 50 epochs, and stabilized after 100 epochs, showing a stable convergence process. Compared with the other two learning rates, this learning rate has the most training epochs, ensuring that the model has enough training time. The final training loss value is similar to that when the learning rate is 0.005, both reaching a smaller value and more stable. The performance at this learning rate is the most ideal, because it ensures sufficient training of the model and a smaller training loss value while maintaining a faster convergence speed. In addition, a stable convergence process also helps improve the generalization ability of the model.

**Learning Rate is 0.00005:** The training loss of the CFMM method on the three data sets decreases very slowly, and the training loss value only decreases by about 20 within 100 epochs. Due to the slow convergence speed, the algorithm stops training around 100 epochs. The final training loss value is larger than that of the other two learning rates, indicating that the model has poor learning effect at this learning rate.

In summary, when the learning rate is 0.0005, the CFMM method performs best on the three data sets. It can maintain a faster convergence speed while ensuring sufficient training of the model and a smaller training loss value.

#### Recall analysis of validation set.

The following is a detailed analysis and conclusion summary of the validation set Recall performance of CFMM under three different learning rates in Fig 5.

**Learning Rate is 0.005:** On larger datasets (such as Clothing and Sports), the recall of the CFMM algorithm on the validation set initially showed an upward trend, then dropped sharply after reaching a peak, almost returning to the initial level. This shows that the learning rate caused the model to quickly learn some features at the beginning of training, but then overfitting occurred, resulting in a sharp drop in performance on the validation set. On small-scale datasets (such as Baby), the recall of the validation set has been showing a downward trend, indicating that the learning rate may be too high for small-scale datasets, resulting in the inability of the model to learn effectively.

**Learning Rate is 0.0005:** On all datasets (Clothing, Sports, Baby), the recall performance of the CFMM algorithm on the validation set is relatively stable and good. For larger datasets, the recall of the validation set quickly rebounds after an initial decline and reaches a stable state; for smaller datasets, the recall of the validation set rises slowly and reaches a stable state. In all cases, this learning rate makes the recall of the validation set reach a level that other learning rates have not reached, indicating that the model under this learning rate has better generalization ability.

**Learning Rate is 0.00005:** On all datasets, the recall of the CFMM algorithm on the validation set was initially low, but then rose rapidly within 50 epochs. However, after reaching the highest point, the recall of the validation set began to slowly decline on the larger datasets, while it gradually leveled off on the smaller datasets. This may be due to the low learning rate, which caused the model to learn too slowly and fail to fully converge to the optimal solution during training.

In summary, when the learning rate is 0.0005, the CFMM algorithm has the best Recall performance on the validation set, is relatively stable and has good generalization ability.

### Comparison with and without fusion loss

**Fig 6:**
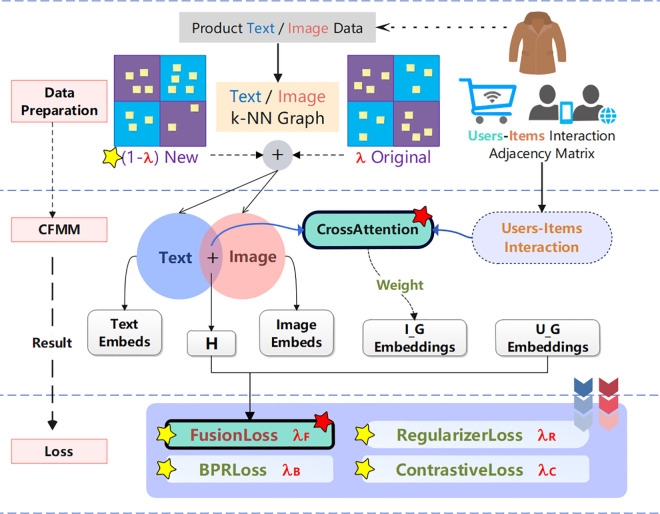
Position display of different modules and parameters. The red stars mark the specific locations of the Cross-Attention and Fusion Loss modules, and the yellow stars mark the specific locations of the four loss parameters and the Lambda coefficient.

To verify the impact of Fusion Loss proposed in this study on CFMM performance, experiments were conducted on three widely used real-world datasets (Clothing, Sports, and Baby), and the performance of the model was tested with and without Fusion Loss. As a key innovation of CFMM, Fusion Loss aims to further improve the accuracy of recommendation systems by optimizing the multimodal feature fusion process. Its location is shown in Fig 6. The evaluation indicators used were Recall@K and NDCG@K, where K was set to 20 to evaluate the performance of the model at the top of the recommendation list.

The experimental results are shown in Table 2, which shows the Recall@20 and NDCG@20 scores obtained by the CFMM model with and without Fusion Loss on three datasets.

**Table 2:** Comparison of CFMM performance with and without Fusion Loss, with and without Cross-Attention, and with different parameters.

Performance comparison of different models	Clothing		Sports		Baby	
	Recall@20	NDCG@20	Recall@20	NDCG@20	Recall@20	NDCG@20
No Fusion Loss	0.0795	0.0355	0.1012	0.0465	0.0892	0.0404
**Have** Fusion Loss	0.0796	0.0355	0.1012	0.0465	0.0897	0.0407
No Cross-Attention	0.0788	0.0354	0.1008	0.0464	0.0904	0.0409
**Have** Cross-Attention	0.0796	0.0355	0.1012	0.0465	0.0897	0.0407
*λ*_*F*_ = 1*10^-3^	0.0795	0.0358	0.1008	0.0463	0.0896	0.0409
*λ*_*F*_ = 1*10^-5^	0.0796	0.0355	0.1012	0.0465	0.0897	0.0407
*λ*_*F*_ = 1*10^-7^	0.0796	0.0356	0.1012	0.0464	0.0896	0.0408
*λ*_*B*_ = 0.1	0.0716	0.0318	0.0929	0.0416	0.0816	0.0363
*λ*_*B*_ = 1	0.0796	0.0355	0.1012	0.0465	0.0897	0.0407
*λ*_*B*_ = 10	0.0742	0.0339	0.0978	0.0446	0.0842	0.0377
*λ*_*R*_ = 1*10^-3^	0.0794	0.0355	0.1004	0.0460	0.0890	0.0400
*λ*_*R*_ = 1*10^-5^	0.0796	0.0355	0.1012	0.0465	0.0897	0.0407
*λ*_*R*_ = 1*10^-7^	0.0795	0.0355	0.1013	0.0462	0.0892	0.0405
*λ*_*C*_ = 0.3	0.0722	0.0320	0.0925	0.0413	0.0816	0.0361
*λ*_*C*_ = 0.03	0.0796	0.0355	0.1012	0.0465	0.0897	0.0407
*λ*_*C*_ = 0.003	0.0742	0.0338	0.0982	0.0448	0.0848	0.0380
λ = 0.2	0.0796	0.0354	0.1009	0.0466	0.0903	0.0406
λ = 0.5	0.0800	0.0359	0.1017	0.0467	0.0889	0.0397
λ = 0.8	0.0796	0.0355	0.1012	0.0465	0.0897	0.0407

*λ*_*F*_, *λ*_*B*_, *λ*_R_, *λ*_*C*_ and λ are the parameters of Fusion, BPR, Regularization, Contrastive and Lambda Coefficient respectively.

The results demonstrate that the introduction of Fusion Loss improves the performance of the CFMM model across all three datasets. Specifically, for the Clothing dataset, Recall@20 increased by 0.16% and NDCG@20 increased by 0.06% when Fusion Loss was included. For the Sports dataset, with Recall@20 increasing by 0.05% and NDCG@20 increasing by 0.13%. Similarly, for the Baby dataset, Recall@20 and NDCG@20 improved by 0.56% and 0.74%, respectively. These improvements suggest that Fusion Loss effectively optimizes the multimodal feature fusion process, enabling the CFMM model to generate more accurate and relevant recommendations.

In summary, the experimental results provide evidence that the Fusion Loss function proposed in this paper plays a crucial role in enhancing the performance of the CFMM model. On large-scale datasets, Fusion Loss can significantly improve the quality of fused features by optimizing the fusion process of multimodal features. Due to the large amount of data, the model has more opportunities to learn effective fusion methods between different modal features, thereby generating more accurate and useful fusion feature representations. On small-scale datasets, the model is prone to overfitting to limited data. Fusion Loss introduces the concept of relative ranking to constrain the ranking relationship of fused features, which helps prevent the model from overfitting to the training data and improves the model’s generalization ability on unseen data. By integrating Fusion Loss into the model, we are able to achieve significant improvements in both recall rate and ranking precision on multiple real-world datasets.

### Comparison with and without cross-attention

In this section, we explore the performance differences of the CFMM model with and without the Cross Attention module to comprehensively evaluate the impact of cross-attention on model recommendation performance. Its location is shown in Fig 6. The experimental results are based on three widely used real-world datasets: Clothing, Sports, and Baby. Recall @K and NDCG@K were used as evaluation indicators, with K set to 20, to evaluate the model performance.

The Cross-Attention module can capture the interactive relationship between the multimodal features of items and users, thereby achieving more effective feature fusion and recommendation, which has a positive impact on the performance of CFMM on the Closing and Sports datasets. Therefore, in large-scale data sets, due to the huge amount of data, product information usually contains multiple modalities, and the information within each modality is also very rich. The Cross-Attention mechanism can make full use of these rich multimodal features, and achieve deep feature fusion by calculating the attention weights between different modal features, thereby capturing more complex and detailed user preferences and product characteristics. However, the performance on the Baby dataset suggests that in practical applications, the model structure and parameter settings should be flexibly adjusted according to the characteristics of the dataset to achieve the best recommendation effect. The applicability of the two modules in the recommendation system is reflected in the deepening of multimodal feature interaction and the optimization of feature extraction, which is also demonstrated by the experimental results.

The experimental results are shown in Table 2, the results show that on large-scale datasets (such as Clothing and Sports), the CFMM model with the cross-attention mechanism improves both the Recall@20 and NDCG@20 indicators. For the Clothing dataset, after adding Cross-Attention, Recall@20 increased by 1.00% and NDCG@20 increased by 0.25%; for the Sports dataset, Recall@20 increased by 0.39% and NDCG@20 increased by 0.22%. This further verifies the effectiveness of cross-attention in processing complex datasets, especially in improving the accuracy and ranking capabilities of recommendation systems.

Notably, on the Baby dataset, the Cross-Attention module did not improve Recall@20 and NDCG@20, but decreased slightly. This may be due to the limited data volume of the Baby data set, as well as related data characteristics and distribution. This result shows that the Cross-Attention module may perform differently on different types of data sets and needs to be adjusted and optimized according to the characteristics of the specific data sets.

In summary, the Cross-Attention module can significantly improve the recommendation performance of the CFMM model; however, the performance differs on different datasets. In the future, more in-depth research should be conducted on optimization and adjustments based on the characteristics of specific datasets to further improve the accuracy and efficiency of recommendation systems.

### Comparison of different fusion loss parameters

In order to deeply explore the specific impact of Fusion Loss parameter settings *λ*_*F*_ on the performance of the CFMM model, we conducted detailed experiments on three datasets: Clothing, Sports, and Baby, and tested the model performance under different Fusion Loss parameters (1*10^−3^, 1*10^−5^, 1*10^−7^), aiming to fully explore the impact of parameters on model performance and verify the effectiveness of Fusion Loss under different settings. The location of this parameter is shown in Fig 6.

As shown in Table 2, in the key parameter tuning strategy section, the focus is mainly on the optimization selection of five core parameters. We carefully set the parameters of BPR Loss, Regularization Loss, and Contrastive Loss based on the previous studies and model characteristics. To enhance the influence of original data in model updates, we set the Lambda Coefficient to fuse the original features with the new features at a ratio of 8:2 to ensure stability during model updates. When determining the Fusion Loss weight, we tested different parameter ranges and finally selected 1 × 10^−5^, which performed best on most data sets, to effectively fuse multimodal features.

**Fig 7:**
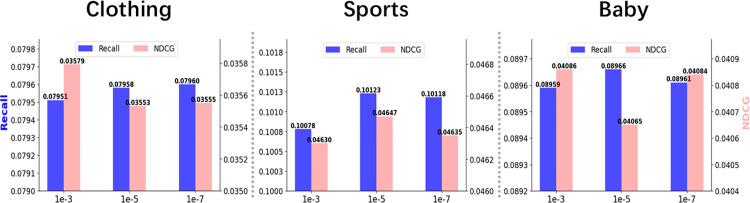
Performance comparison of different Fusion Loss parameters. Impact of different Fusion Loss parameter settings on Recall@20 and NDCG@20 performance metrics on the Clothing, Sports, and Baby datasets.

The experimental results are shown in Table 2 and Fig 7, where the blue histogram in Fig 7 represents the Recall@20 indicator and the red histogram represents the NDCG@20 indicator.

#### Recall@20 comparison.

In terms of the Recall@20 indicator, the experimental results clearly show that the Fusion Loss parameter has a significant effect on the recall rate of the model.

Specifically, Clothing dataset: Recall@20 reached the highest value of 0.0796 when the Fusion Loss parameter was set to 1*10^−5^. This is an improvement compared with the 1*10^−3^ parameter settings.

Sports dataset: Similarly, when the Fusion Loss parameter was 1*10^−5^, Recall@20 reached the maximum value of 0.1012.

Baby dataset: Although the changes in the Recall value under different Fusion Loss parameters are relatively small, the 1*10^−5^ parameter still achieved the best performance (0.0897).

#### NDCG@20 analysis.

For the NDCG@20 metric, the experimental results show some interesting findings:

Clothing dataset: Unlike Recall@20, NDCG@20 did not reach its maximum value when the Fusion Loss parameter was 1*10^−5^, but reached its maximum value (0.0358) when it was 1*10^−3^, indicating that a larger Fusion Loss parameter (such as 1*10^−3^) on the Clothing dataset may be more conducive to optimizing the sorting accuracy.

Sports dataset: In the Sports dataset, when the Fusion Loss parameter is 1*10^−5^, NDCG@20 reaches its maximum value of 0.0465, indicating that this parameter setting significantly improves the sorting accuracy on this dataset.

Baby dataset: In the Baby dataset, when the Fusion Loss parameter is 1*10^−5^, the NDCG@20 value is not the highest but the lowest (0.0407) among all test parameters. This phenomenon may be caused by the fact that the Baby dataset has unique data distribution characteristics, which makes certain model parameter settings not always optimal for this dataset. Compared with the Clothing and Sports datasets, the Baby dataset may have fewer features, so the setting of the Fusion Loss parameters needs to closely match the characteristics of the dataset to maximize the sorting quality.

#### Comprehensive analysis.

The selection of the Fusion Loss parameters has a significant impact on model performance, and in most cases, appropriately reducing the Fusion Loss parameter (such as 1*10^−5^) can improve the recall rate of the model (Recall@20). However, in terms of ranking accuracy (NDCG@20), the optimal parameters may vary depending on the characteristics of the dataset.

### Comparison of CFMM using different parameters

**Fig 8:**
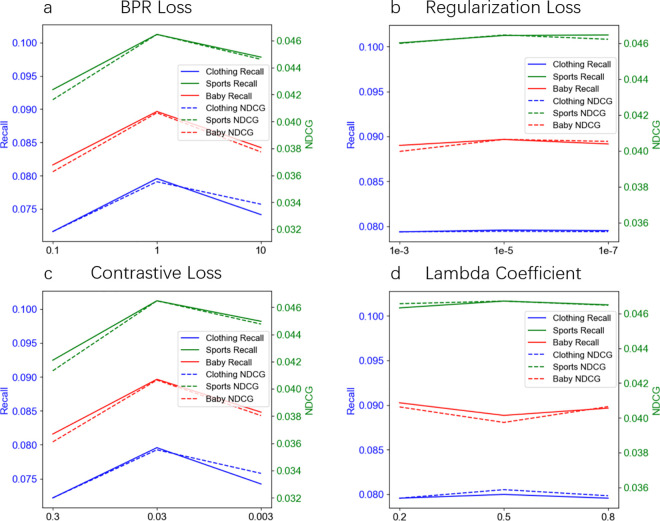
Performance comparison of CFMM using different hyperparameters. This figure shows the Recall and NDCG performance indicators of the CFMM model on the Clothing (Blue), Sports (Green) and Baby (Red) datasets when using different hyperparameters of the BPR (a), Regularization (b), Contrastive (c) Loss function and different Lambda Coefficient (d). The solid line in the figure represents the Recall value using the current dataset, and the dotted line represents the NDCG value using the current dataset. The horizontal axis represents different parameters or coefficients, and the vertical axis corresponds to the Recall and NDCG values respectively.

Through comparative experiments using different parameters on the CFMM model, the study found that parameters such as BPR Loss, Regularization Loss, Contrastive Loss, and the Lambda coefficient in the feature map skip connection have a significant impact on model performance, as shown in Table 2 and Fig 8. The location of these parameters is shown in Fig 6. Choosing appropriate parameter settings can optimize the recommendation performance of the model and achieve better performance on Recall, NDCG and other indicators. These findings provide valuable reference for parameter tuning of CFMM models in practical applications.

#### Different BPR loss parameters.

It can be clearly seen from Table 2 and Fig 8 (a) that when the BPR Loss *λ*_*B*_ is set to 1 parameter, the Recall and NDCG values of the CFMM model on the three data sets reach the highest.

Experimental results show that when BPR Loss is 1, the recall and ranking capabilities of the model can be well balanced. Too small a loss parameter (such as 0.1) may cause the model to underfit the training data, while too large a loss parameter (such as 10) may cause the model to overfit the training data and reduce generalization ability. When the parameter is 1, it is at a better balance point, which not only ensures the fitting effect of the model but also avoids the risk of overfitting. Different data sets have different data distributions and characteristics, and when the parameter is 1, it can show good performance on the three data sets of Clothing, Sports and Baby, which shows that this parameter setting has certain universality and data adaptability.

#### Different regularization loss parameters.

It can be clearly seen from Table 2 and Fig 8 (b) that when the Regularization Loss parameter *λ*_*R*_ is set to 1*10^−5^, the Recall and NDCG values of the CFMM model on the three data sets are the highest.

Experiments show that this parameter setting (1*10^−5^) can more effectively optimize the recommendation performance of the model and enable the model to better capture the potential relationship between users and products. A too small Regularization Loss parameter (such as 1*10^−7^) may cause the model to be too complex and easy to overfit the training data; while a too large Regularization Loss parameter (such as 1*10^−3^) may inhibit the learning ability of the model and lead to performance degradation. As a moderate value, 1*10^−5^ can achieve a good balance between model complexity and performance.

#### Different contrastive loss parameters.

It can be clearly seen from Table 2 and Fig 8 (c) that when the Contrastive Loss parameter *λ*_*C*_ is set to 0.03, the Recall and NDCG values of the CFMM model on the three data sets reach the highest.

Contrastive Loss is a loss function used to enhance the difference between positive and negative samples, especially when dealing with multimodal data. In the CFMM model, Contrastive Loss is used to constrain the fusion of multimodal information, thereby improving the performance of the recommendation system. Too large a parameter value (such as 0.3) will make the model pay too much attention to the difference between positive and negative samples, causing the model to be too sensitive during training and prone to overfitting, especially on datasets with small data volumes. Too small a parameter value (such as 0.003) will make the model insensitive when distinguishing between positive and negative samples, resulting in the difference between positive and negative samples not being obvious enough, thus affecting the recommendation performance of the model. When the parameter value is 0.03, the model can maintain a moderate degree of distinction between positive and negative samples, neither too strict nor too loose, which is conducive to learning better feature representations. This shows that a moderate Contrastive Loss parameter setting helps the model maintain a moderate degree of distinction between positive and negative samples, thereby improving the overall performance of the recommendation system. Therefore, using a Contrastive Loss parameter of 0.03 in the CFMM model is a more reasonable choice.

#### Different lambda coefficient of skip connection.

Lambda Coefficient is a hyperparameter that controls the mixing ratio of the original feature map and the newly generated feature map in the skip connection. It is used in the update process of image and text feature maps to achieve smooth update of feature maps by weighting the original feature map and the feature map calculated based on the new similarity matrix. When the value of Lambda Coefficient is close to 0, the newly calculated feature map dominates the update process and the original feature map has less influence. When the value of Lambda Coefficient is close to 1, the original feature map dominates the update process and the newly calculated feature map has less influence, the experimental results are shown in Table 2 and Fig 8 (d).

In the CFMM model, Lambda Coefficient (λ) is a key hyperparameter used to balance the information fusion ratio between the original feature map and the map obtained through training. Although from the perspective of a single dataset, λ = 0.5 performs best on the Clothing and Sports datasets, λ = 0.8 performs best on the Baby dataset. In order to select a comprehensive optimal λ value that is suitable for all datasets, we need to consider the overall generalization ability and stability of the model. Selecting a λ value that performs well on all datasets helps ensure the stability of the model. λ = 0.8 has less performance fluctuations on the three datasets, showing higher stability. Therefore, considering the generalization ability and stability comprehensively, we can consider λ = 0.8 to be a better choice.

## Conclusion

The CFMM model significantly enhances recommendation performance by deeply integrating multimodal information and optimizing feature fusion. It introduces a Cross-Attention mechanism for deep multimodal feature fusion and a comprehensive loss function for training optimization. Extensive experiments on three datasets show CFMM outperforms baselines, verifying its effectiveness and generalization. Key component analysis further highlights the importance of Cross-Attention and the loss function design. Future work will explore CFMM’s applicability in more domains.

Although the CFMM algorithm shows potential in multimodal data processing and has moderate algorithm complexity, its performance is highly dependent on data quality and is easily affected by noise and missing data. Its performance on data sets of different sizes varies slightly. At the same time, the algorithm training requires good hardware support and a lot of experiments. In addition, its internal mechanism is opaque and its interpretability is limited, which limits its application in some scenarios. Future research needs to optimize these challenges.
